# Prediction of Potential Biomarkers in Early-Stage Nasopharyngeal Carcinoma Based on Platelet RNA Sequencing

**DOI:** 10.1007/s12033-022-00611-z

**Published:** 2022-11-29

**Authors:** Yuanji Xu, Lin Chen, Yijian Chen, Wangzhong Ye, Xinyi Huang, Mingyao Ke, Guodong Ye, Liancheng Lin, Kangmei Dong, Zhizhong Lin, Penggang Bai, Chuanben Chen

**Affiliations:** 1 Department of Radiation Oncology, Clinical Oncology School of Fujian Medical University, Fujian Cancer Hospital, No.420, Fuma Road, Fuzhou, 350014 Fujian China; 2 Cancer Center, West China Xiamen Hospital of Sichuan University, Xiamen, Fujian China; 3 Institute of Respiratory Diseases, Xiamen Medical College, Xiamen, Fujian China; 4 Fujian Collaborative Innovation Center for Accurate Medicine of Respiratory Diseases, Xiamen, Fujian China; 5 Xiamen LifeInt Technology Co., Ltd, Xiamen, Fujian China; 6 Innovation Center for Cancer Research, Clinical Oncology School of Fujian Medical University, Fujian Cancer Hospital, Fuzhou, Fujian China

**Keywords:** Nasopharyngeal carcinoma, Tumour-educated blood platelets, Differentially expressed mRNAs, Differentially expressed lncRNAs, Biomarkers

## Abstract

Early diagnosis is essential for the treatment and prevention of nasopharyngeal cancer. However, there is a lack of effective biological indicators for nasopharyngeal carcinoma (NPC). Therefore, we explored the potential biomarkers in tumour-educated blood platelet (TEP) RNA in early NPC. Platelets were isolated from blood plasma and their RNA was extracted. High-throughput sequenced data from a total of 33 plasma samples were analysed using DESeq2 to identify the differentially expressed genes (DEGs). Subsequently, the DEGs were subjected to principal component analysis (PCA), gene ontology (GO) analysis, and Kyoto Encyclopedia of Genes and Genomes (KEGG) pathway enrichment analysis; and Cytoscape, TargetScan, and miRanda software were used for inferring the competing endogenous RNA network. We identified 19 long non-coding (lnc) RNAs (DElncRNAs) and 248 mRNAs (DEmRNAs) that were differentially expressed in the TEP RNA. In addition, SELP gene mRNA and lncRNAs AC092135.3, AC012358.2, AL021807.1, AP001972.5, and GPX1 were found to be down-regulated DEmRNA and DElncRNAs in the early stage of NPC. Bioinformatic analysis showed that these DEmRNAs and DElncRNAs may be involved in regulating the pathogenesis of NPC. Our research may provide new insights for exploring the biological mechanisms of NPC and early diagnosis using potential biomarkers.

**Supplementary Information:**

The online version contains supplementary material available at 10.1007/s12033-022-00611-z.

## Introduction

Nasopharyngeal carcinoma (NPC) is a highly metastatic and aggressive malignant tumour of the nasopharyngeal epithelial cells [1]. In 2018, about 129,000 people were diagnosed with NPC, among which, the mortality rate was as high as 56.6% [2]. According to reports, the 5-year survival rate of early NPC is significantly higher than that of advanced patients, with a rate close to 100% for stage I patients and only 70.5% for patients in stage IV [3]. Unfortunately, due to the insidious early symptoms of nasopharyngeal cancer, most patients are already in the advanced stage when they are diagnosed [4]. Therefore, early diagnosis of NPC is very important to improve the survival rate [5], but there is still a lack of effective biological indicators for the early diagnosis of NPC.

In recent years, liquid biopsy has attracted increasing attention as a non-invasive cancer detection method [6]. The currently used blood-derived biomarkers include circulating free DNA (cfDNA), circulating tumour DNA (ctDNA), circulating tumour cells (CTC), micro RNA (miRNA), and exosomes [7]. Since cfDNA, ctDNA, and CTC rely on substances released by cancer cells into the peripheral blood stream, these cannot be used for a timely diagnosis in the early stage of cancer. Platelet RNA, however, comes into contact with tumours through the systemic blood circulation, and is present in all stages of tumour occurrence. Both in theory and practice, it can make up for the deficiency of the existing liquid biopsy technique in early cancer diagnosis.

Previous reports suggest that the method of platelet mRNA sequencing can be used to diagnose cancer (i.e. non-small lung cancer, colorectal cancer, glioblastoma, pancreatic adenocarcinoma, and hereditary breast cancer), and the diagnosis accuracy rate can reach 71% [8]. The interaction between tumour cells and platelets has been shown to lead to a specific expression of mRNA in platelets [9, 10]. The RNA levels in TEPs are different from those of healthy platelets. There are many types of platelet RNAs, such as mRNA and long non-coding RNA [11]. Long non-coding RNAs (lncRNAs) are more than 200 nucleotides long and are involved in RNA interference (RNAi) [12, 13]. There is evidence that lncRNA disorders are involved in cell transformation and development of a variety of cancers, including NPC [14, 15]. Through microarray and high-throughput RNA sequencing, a large number of lncRNAs have been identified in NPC tissues and cell lines [16]. One of the main mechanisms for tumours to “educate” platelets is through tumour-derived extracellular vesicles, which are captured by the platelets and ultimately lead to abnormal RNA profiles, including those of lncRNAs [17]. Three novel TEP biomarkers for lung cancer diagnosis and prediction of progression have been reported: lnc‑GTF2H2‑1, RP3‑466P17.2, and lnc‑ST8SIA4‑12 [11]. These encouraging results indicate that TEP RNA could have a great potential as a biological indicator for early diagnosis of NPC.

In this study, we aimed to elucidate the molecular mechanisms of TEP RNA in the early diagnosis of NPC and determine effective biomarkers. Platelet RNA was extracted from healthy donors and patients with early and advanced NPC. Platelet RNA sequencing was combined with machine learning algorithms (TEPseq) and gene expression database (GEO) analysis, to explore simple and effective blood tumour biomarkers for early diagnosis of NPC. Our research can increase the early diagnosis rate of this disease and significantly improve the survival prognosis of patients.

## Materials and Methods

### Blood Sample Collection

Blood samples were collected from 11 patients with early and 11 patients with advanced NPC, admitted to Fujian Cancer Hospital between July and November 2020. Normal blood samples came from 11 healthy blood donors. The Ethics Committee of Fujian Cancer Hospital approved the use of the human tissue specimens related to this work (Project Ethics Number: SQ2019-018-01) and informed consent was obtained. The research method meets the standards set by the Declaration of Helsinki.

### Library Preparation and Sequencing

The whole blood was slowly transferred to a 15-mL centrifuge tube and centrifuged at 400 × *g* at room temperature (25 °C) for 10 min. A pipette was used to transfer the platelet-containing plasma to a 1.5-mL centrifuge tube, which was centrifuged at 360 × *g* for 20 min at room temperature (25 °C). After centrifugation, the supernatant was discarded, and 50 μL of 1 × PBS was added to resuspend the platelets. All the isolated platelets were used for RNA extraction. We used 4 μL of platelet RNA for micro-amplification to obtain an amplified complementary DNA (cDNA) product after purification. Platelet cDNA (1 ng) was used for library construction, and an RNA library was obtained after purification. The sequencing platform used was NovaSeq 6000 (Illumina, Inc., San Diego, CA, USA), with the PE150, 6G Raw Base sequencing strategy.

### Data Processing and Analysis

First, the raw data of high-throughput sequencing were analysed and converted into raw reads; fastp v0.11.7 (https://github.com/OpenGene/fastp) was used for quality control to filter low-quality data and obtain clean reads. Second, we used the STAR v2.5.4b software to carry out a comparative analysis based on a reference genome. Principal component analysis (PCA) was performed using DESeq2 in R (version 1.34.0) (http://www.r-project.org/) based on gene expression information. The more similar the composition of the sample, the smaller the distance reflected in the PCA chart. The change in the degree of expression for each gene can be obtained through DESeq2 (see “mRNA.expression.csv”). The genes with adjusted *p* value (padj) < 0.05 and fold change |log2FC|≥ 1 were screened as significant DEGs.

### GO and KEGG Enrichment Analysis

The DEGs were mapped in accordance with the Gene Ontology (GO) database (http://www.geneontology.org/) to achieve a rough understanding of their biological functions, pathways, and cellular locations. We performed an analysis through the Kyoto Encyclopedia of Genes and Genomes (KEGG, the main public database on Pathway) to find the pathways that were significantly enriched. Here, significance for enrichment was set at padj < 0.05.

### Gene Interaction Analysis

To study their interactions, we selected the DEGs in the normal *versus* advanced-stage groups to draw a network diagram. We used the MCODE plug-in of the Cytoscape v 3.8.2 software to draw separate network diagrams for the three modules.

### Analysis of lncRNAs Regulation of TEP Genes

The difference analysis of lncRNAs from the sequencing data was carried out with a threshold of |FC|> 1.5 and padj < 0.05. The differential expression of lncRNAs and mRNAs from the three groups was analysed to establish the correlation between the Fragments Per Kilobase Million (FPKM) value (log2 logarithm) of each lncRNA and mRNA in the 33 samples. According to the up-regulating or down-regulating relationship and the correlation of the DEGs in the normal *versus* advanced-stage groups, the network was narrowed down using the *r* > 0.9, *p* < 0.01 parameters to find highly regulated lncRNA–mRNA molecules. We checked the relevant literature to further screen the lncRNA–mRNA relationship with platelet-regulated genes. The mRNA in the co-expression network underwent GO and KEGG enrichment analyses to reveal the main functions of the network.

### Competing Endogenous RNA (ceRNA) Network Construction

The lncRNA and mRNA in the co-expression network map were screened out, and all known miRNAs were used. The TargetScan (version 7.2) (http://www.targetscan.org/) database was used to analyse the miRNA–mRNA targeting relationship, while the miRanda software (GPLv2) was employed to predict the lncRNA–miRNA binding relationship; if the minimum free energy for binding showed a value greater than 30, the ceRNA network was drawn.

## Results

### Identification of DEGs in TEPs

**Fig. 1:**
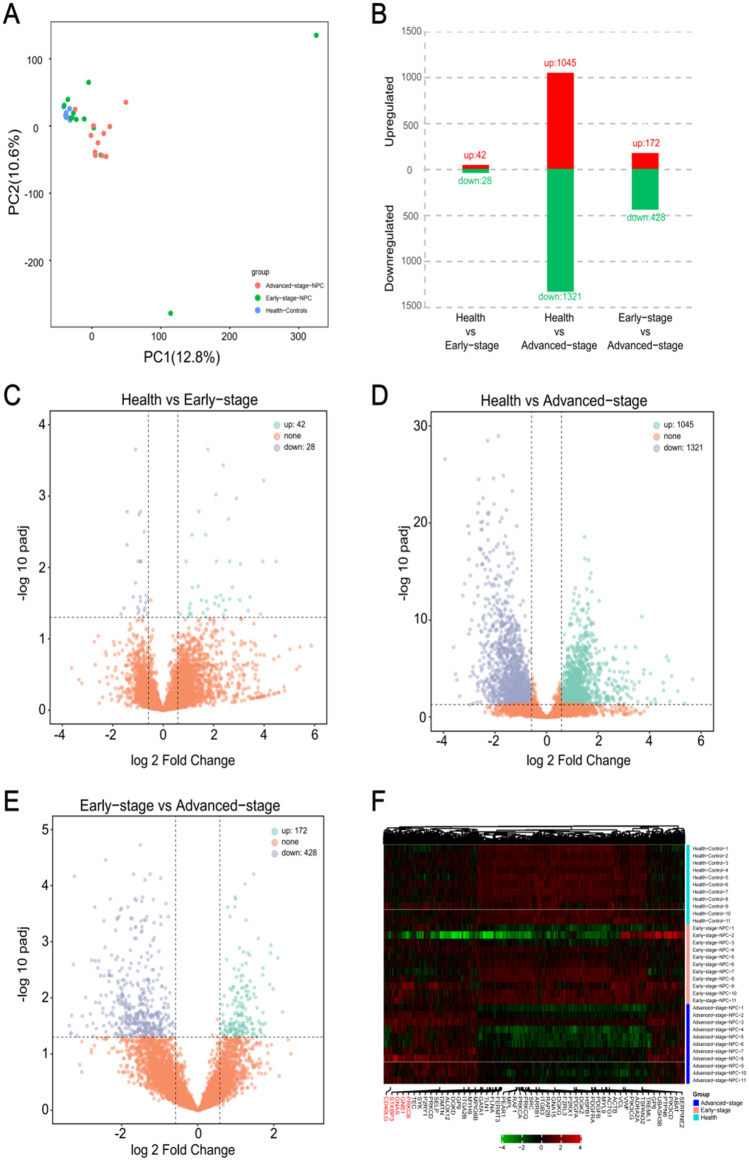
Changes in the gene expression profiles of patients with nasopharyngeal carcinoma (NPC). **A** Principal component analysis (PCA) diagram of the three groups of samples (red indicates patients with advanced nasopharyngeal cancer, green indicates patients with early nasopharyngeal cancer, and blue indicates healthy donors). **B** The number of differentially expressed genes in Normal *versus* Early-stage, Normal *versus* Advanced-stage, Early-stage *versus* Advanced-stage, where the threshold for differential gene screening was |FC| > 1.5 and padj < 0.05. **C**–**E** The volcano map of the differentially expressed genes in Normal *versus* Early-stage (**C**), Normal *versus* Advanced-stage (**D**), and Early-stage *versus* Advanced-stage (**E**). The screening threshold of differential genes satisfied both Log2 Fold Change ≥ 1 and padj ≤ 0.05. **F**: Heat maps of the differentially expressed genes in Normal, Early-stage, and Advanced-stage samples. Red and green colours represent up- and down-regulated genes, respectively. The genes marked red are those that are up-regulated in the advanced group

To determine the rule of sample aggregation and eliminate outliers, we performed a PCA on the mRNA expression profiles between the three groups of samples. We found that there was a partial gene overlap between the normal samples and the early stage of NPC, with only slight differences. However, the advanced stage of NPC can be distinguished clearly from the others. The PC1 value of NPC samples was significantly higher than that of normal samples (Fig. 1A). Based on |FC|> 1.5 and padj < 0.05, the DEGs in healthy donors, and early and advanced NPC patients were identified and plotted in a histogram and a volcano map. Compared with donor samples, 42 genes were up-regulated and 28 were down-regulated in the early stage, whereas 1045 genes were up-regulated and 1321 were down-regulated in the advanced-stage samples. Compared with the early NPC samples, 172 genes were up-regulated and 428 genes were down-regulated in the advanced-stage samples (Fig. 1B–E). A heat map was plotted for the total DEGs in the three comparison groups. The DEGs regulating platelet activation are shown in Fig. 1F. Hierarchical cluster analysis showed that DEGs were different in the three groups of samples; the early stage of NPC showed only slight differences from healthy people, while the advanced stage was significantly different from both the other groups. This shows that compared with the healthy population, the gene expression profile of NPC patients is different, and the difference gradually increases with disease progression.

### Analysis of Functions and Pathways Involved in DEGs

**Fig. 2:**
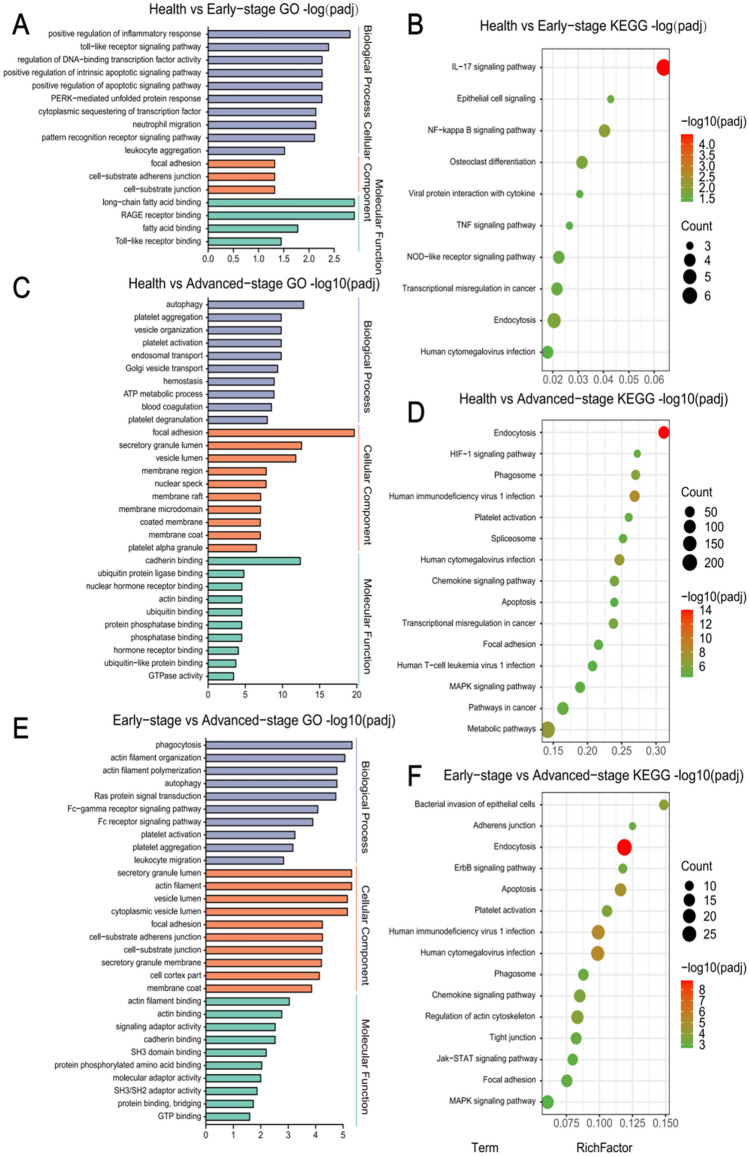
Functional enrichment analysis diagram. **A**, **C**, **E** Bar graph of the GO analysis results of differentially expressed genes in Normal *versus* Early-stage (**A**), Normal *versus* Advanced-stage (**C**), and Early-stage *versus* Advanced-stage (**E**); **B**, **D**, **F** Bubble chart of the KEGG enrichment analysis results of differentially expressed genes in Normal *versus* Early-stage (**B**), Normal *versus* Advanced-stage (**D**), and Early-stage *versus* Advanced-stage (**F**). The threshold for differential gene selection was padj < 0.05

To further research the biological functions of these DEGs, we analysed the target genes through GO and KEGG. GO analysis showed that, compared with healthy people, most of the early-stage DEG functions are relevant to cell connection and positive regulation of intrinsic apoptosis signalling pathways (Fig. 2A); KEGG analysis showed that the NF−kB signalling pathway was mainly enriched (Fig. 2B). Compared with the healthy group, GO analysis revealed that the biological functions of advanced-stage DEGs were closely related to platelet aggregation and activation (Fig. 2C), while the results of the KEGG analysis indicated an enrichment in platelet activation, the MAPK signalling pathway, and cancer-related pathways (Fig. 2D). GO analysis of DEGs in the advanced and early comparison groups showed an enrichment in platelet activation, platelet aggregation, and cell junction (Fig. 2E), while KEGG pathway analysis revealed increased DEGs inherent to platelet activation and MAPK signalling pathways (Fig. 2F).

### Gene Interaction Regulation

**Fig. 3:**
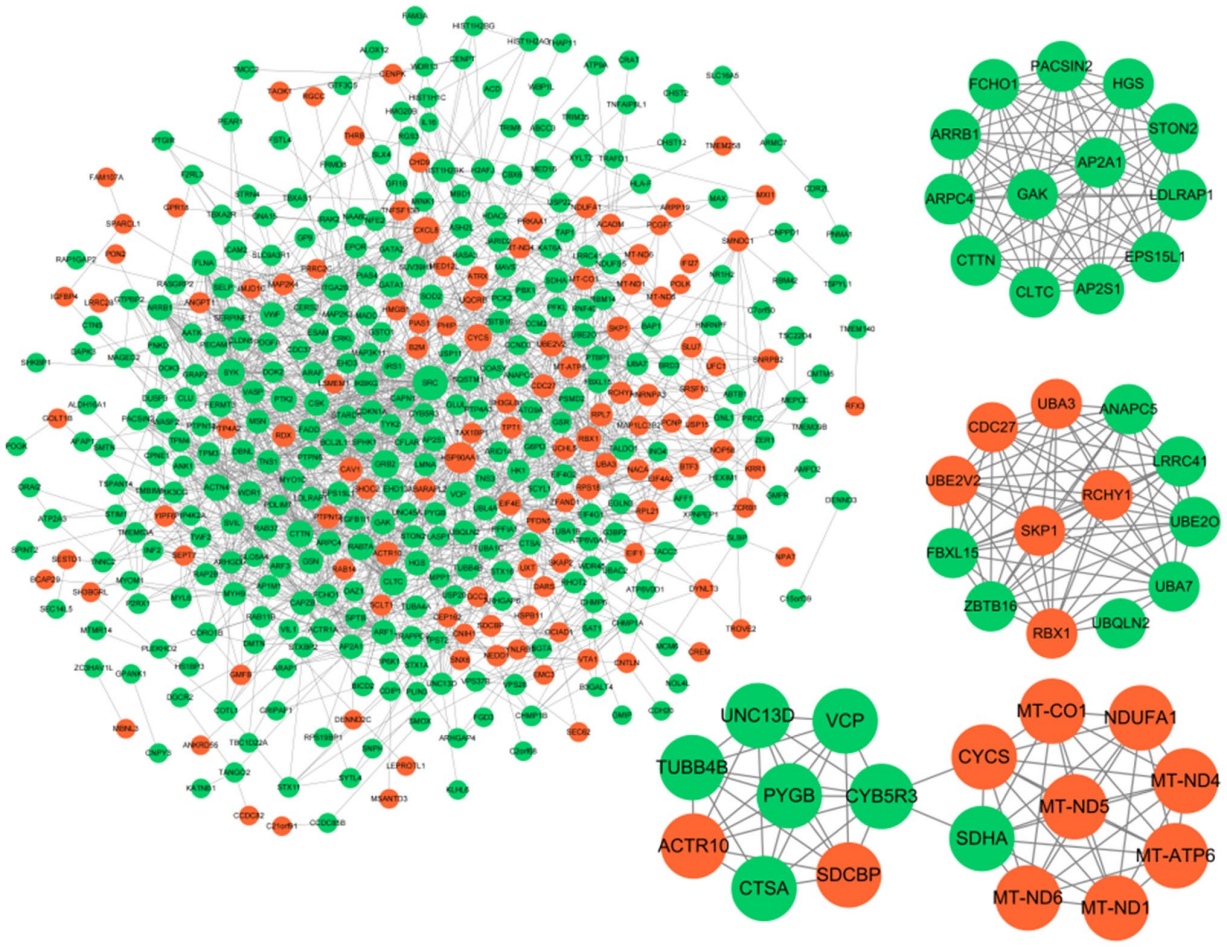
Diagram of interaction networks of differentially expressed genes**.** The diagram of interaction networks of differentially expressed genes (DEGs). The closely regulated modules are taken out separately to draw the network diagram. Up-regulated genes are represented in orange and those down-regulated are represented in green

With the purpose of studying their interactive relationships, we selected the DEGs in the normal *versus* advanced-stage groups to draw a network diagram. As shown in Fig. 3, most genes were found to be down-regulated.

### lncRNA Differential Analysis

**Fig. 4:**
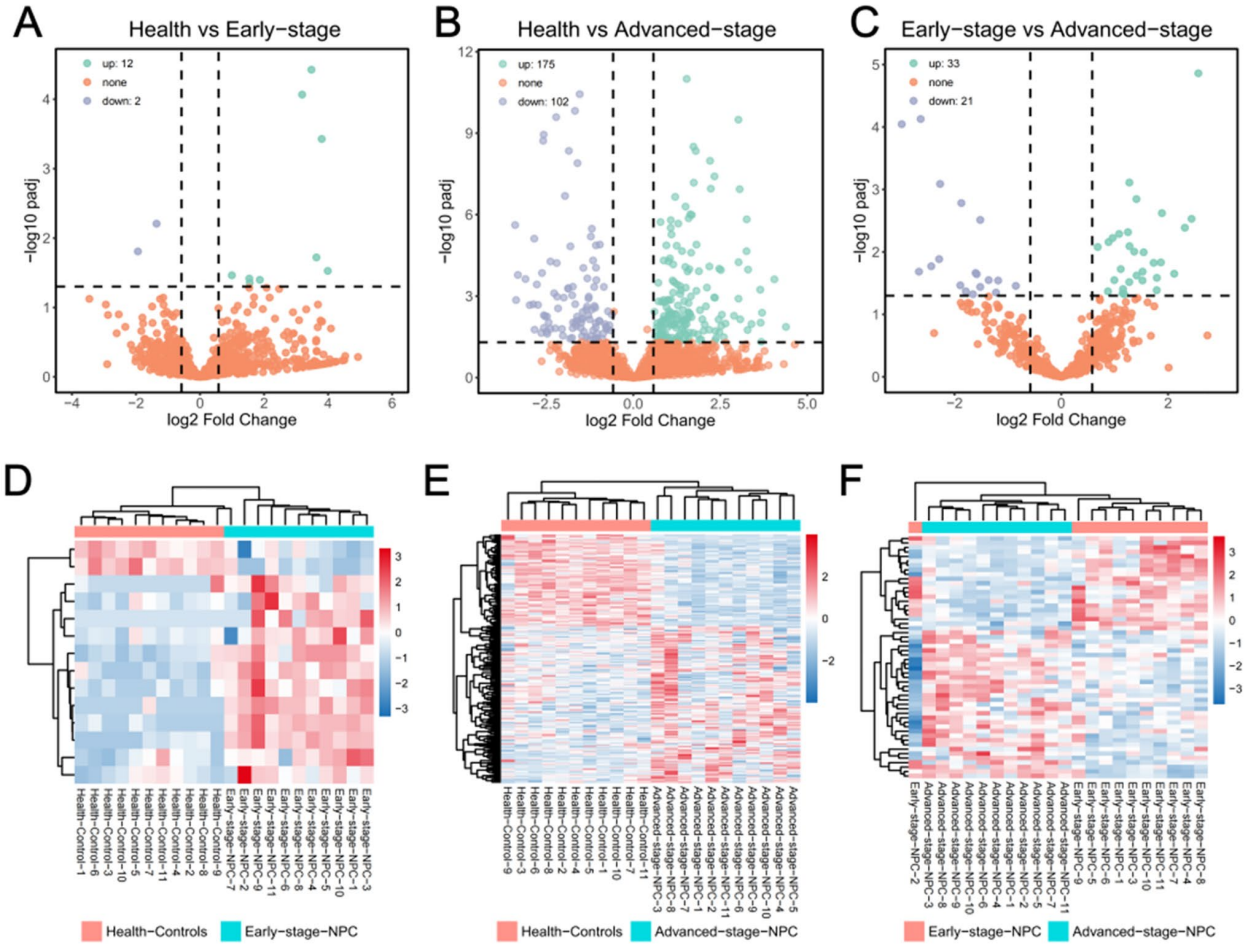
DElncRNAs in the three groups. **A**–**C** Volcano map of DElncRNAs in Normal *versus* Early-stage (**A**), Normal *versus* Advanced-stage (**B**), and Early-stage *versus* Advanced-stage (**C**). Green and blue represent up- and down-regulated genes, respectively, and orange is used for genes that are not differentially expressed. **D**–**F** The heat maps of DElncRNAs in Normal *versus* Early-stage (**D**), Normal *versus* Advanced-stage (**E**), and Early-stage *versus* Advanced-stage (**F**). Red and blue colours represent high and low expression of the gene, respectively

We performed differential analysis on the lncRNA sequencing data. The volcano and heat maps of the DElncRNAs are shown in Fig. 4A–C. Compared with the healthy group, there were 12 early-stage and 175 advanced up-regulated lncRNAs, and 2 early and 102 advanced down-regulated lncRNAs. Compared with the early stage, 33 lncRNAs were up-regulated and 21 were down-regulated in the advanced stage. The heat map of lncRNA expression is shown in Fig. 4D–F. Hierarchical cluster analysis showed that DElncRNAs are present in normal and NPC samples (|FC|> 1.5 and padj < 0.05).

### lncRNAs Regulate TEP Function Genes

**Fig. 5:**
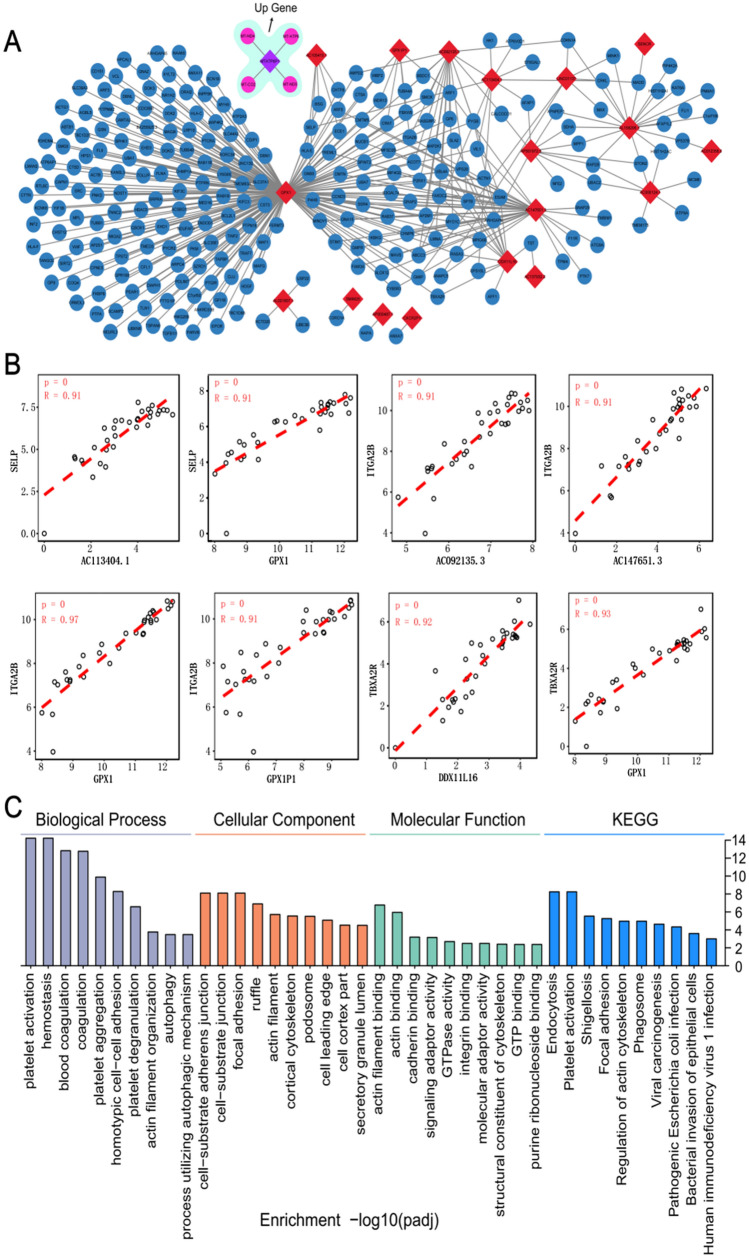
DElncRNA–DEmRNA interactive relationship. **A** The DElncRNA–DEmRNA interaction network in nasopharyngeal carcinoma (NPC); diamonds represent lncRNA, circles represent mRNA. The correlation coefficient of the DElncRNA–DEmRNA pair displayed in the network diagram is > 0.9, and the correlation p value is < 0.01. Most of the genes in the figure are down-regulated, only the up-regulated genes are marked with a single light green background. **B** Scatter plot of the lncRNA–mRNA relationship of platelet-regulated genes. **C** GO and KEGG enrichment analysis of DEmRNAs in Fig. 5A

The lncRNA–mRNA relationship pairs that were concomitantly up- or down-regulated were selected and the correlation network diagram was drawn. There were 163 lncRNAs, 1460 mRNAs, and 39,055 lncRNA–mRNA pairs in the network. Because the network in additional file 1 [Fig. S1] is still too large, only the correlation coefficient > 0.9 and the correlation *p* value < 0.01 were used to draw the network. The smaller network diagram contained 19 lncRNAs, 248 mRNAs, and 352 lncRNA–mRNA pairs (Fig. 5A). As a result, only 1 lncRNA and 4 mRNAs were up-regulated, while the rest were down-regulated.

We screened platelet-regulated genes through the literature and extrapolated a scatter plot with the help of a co-expression network (Fig. 5B). Eight genes are presented in the main text, and six in additional file 2 [Fig. S2]. The results show that SELP and lncRNAs AC113404.1 (*r* = 0.91) and GPX1 (*r* = 0.91) were positively correlated; ITGA2B and lncRNAs AC092135.3 (*r* = 0.91), AC147651.3 (*r* = 0.91), GPX1 (*r* = 0.97), and GPX1P1 (*r* = 0.91) were positively correlated; TBXA2R was positively correlated with lncRNAs DDX11L16 (*r* = 0.92) and GPX1 (*r* = 0.93). GO and KEGG enrichment analyses of 248 DEmRNAs in the co-expression network were performed to gain insights into the underlying biological functions of these mRNAs. As shown in Fig. 5C, GO analysis revealed that (1) for biological processes (BP), these DEmRNAs are mainly enriched in platelet activation, aggregation, and degranulation. (2) For cell components (CC), the cell-substrate junctions, and focal adhesions are significantly enriched. (3) In terms of molecular function, integrin binding is enriched. KEGG analysis revealed that DEmRNAs are particularly abundant in viral carcinogenesis and platelet activation (Fig. 5C). This indicates that these DEmRNAs may be crucial in the process of TEPs promoting the occurrence and development of NPC.

### Construction of a ceRNA Network

**Fig. 6:**
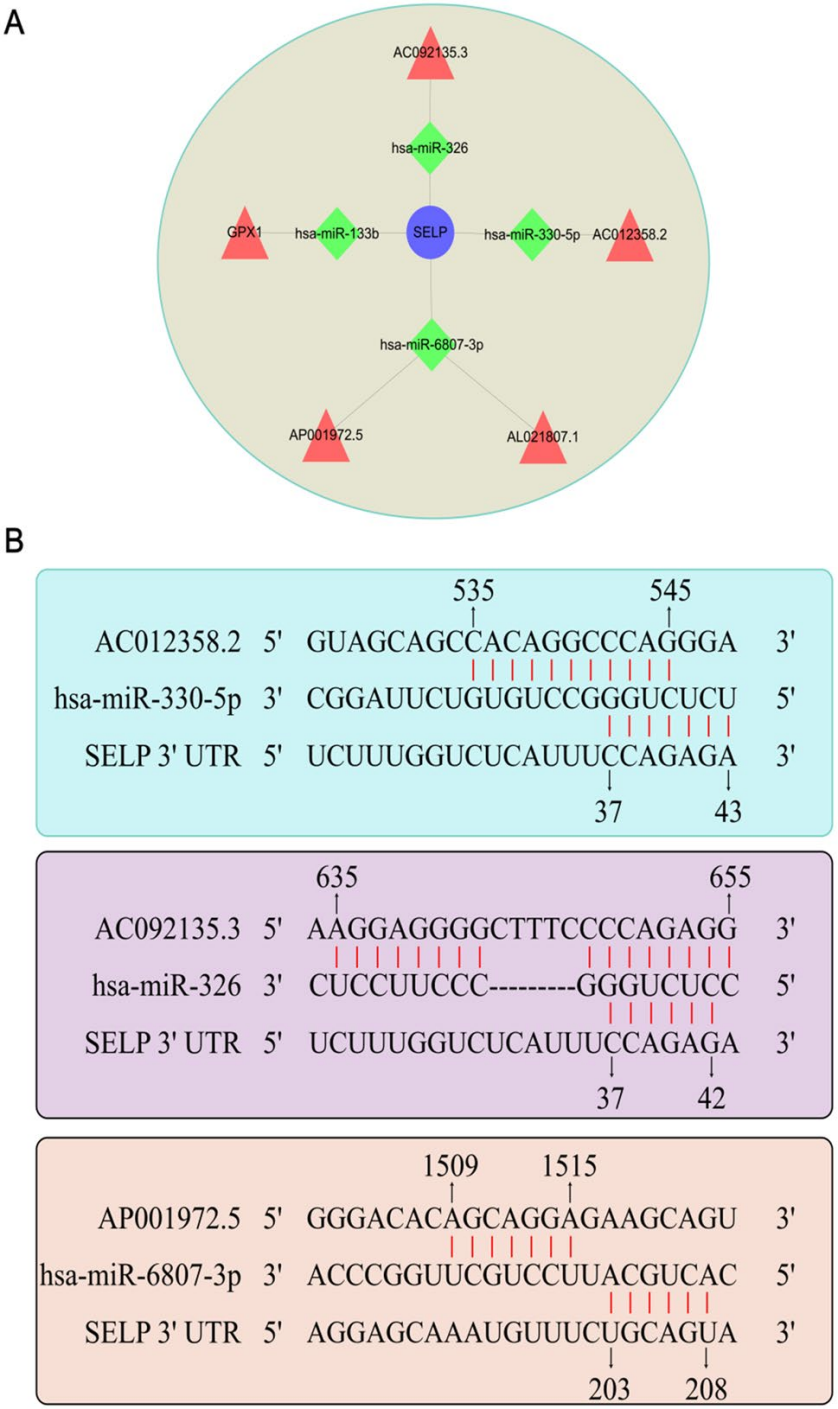
lncRNA–miRNA–mRNA ceRNA network diagram. **A** Diagram of lncRNA–miRNA–mRNA regulation. mRNA is indicated in blue, miRNA in green, and lncRNA in red. There are 5 pathways in total. **B** Binding site map of some of the lncRNA–miRNA–SELP of Fig. 6A

To verify that lncRNAs may regulate mRNAs by regulating miRNAs, we drew a ceRNA network diagram, as shown in additional file 3 [Fig. S3]. It was found that SELP is the only gene in the ceRNA network that overlaps with the target gene. Finally, we built a ceRNA network in NPC based on 5 lncRNAs, 4 miRNAs, and 1 mRNA nodes (Fig. 6A). The base binding sites of SELP-hsa-miR-330-5p-AC012358.2, SELP-hsa-miR-326-AC092135.3, and SELP-hsa-miR-6807-3p-AP001972.5 are shown in Fig. 6B.

## Discussion

Early screening and diagnosis of NPC is of critical importance for improving treatment efficacy [18, 19]. However, accurate early indicators for NPC are still missing. Therefore, there is an urgent need to identify novel diagnostic biomarkers for the early diagnosis of NPC patients [20]. In recent years, TEP RNA has become a research hotspot as a biomarker in tumour detection. It has been reported that platelet RNA can be used as a biomarker to distinguish early liver cancer from nodules of cirrhosis [21]. Sidra Asghar et al. found that AKT expression in platelets of early HCC was reduced by 0.6 times and PI3K expression was reduced by 0.65, indicating a potential as diagnostic markers for early HCC [22]. Yang et al. found that TIMP1 mRNA in platelets of colorectal cancer patients can promote tumour growth and apoptosis, which can be used as an independent diagnostic biomarker [23]. Therefore, the TEP RNA spectrum has potential both for the early diagnosis of NPC and as a biomarker.

The purpose of this study was to investigate the molecular mechanisms of TEP RNA in the early diagnosis of NPC and to determine effective biomarkers. Platelet RNA was extracted from the plasma of early- and advanced-stage NPC patients and healthy donors in our hospital. The co-expressed (concomitantly up-regulated or down-regulated) DEGs identified include 19 lncRNAs and 248 mRNAs. Among them, SELP along with AC092135.3, AC012358.2, AL021807.1, AP001972.5, and GPX1 seem as important DEmRNA and DElncRNAs in the early stage of NPC. The analysis of the sequencing data showed that the down-regulation of SELP shows little difference between early NPC and healthy individuals, while an obvious difference is found in advanced NPC, which gradually increases from the early to the advanced stage. The down-regulation of SELP and AC092135.3, AC012358.2, AL021807.1, AP001972.5, and GPX1 promotes the progression of NPC.

The SELP gene (also referred to as CD62, GRMP, and PSEL) is located on chromosome 1q21-q24, which can encode p-selectin [24]. P-selectin belongs to the selectin family (P-, L- and E-selectins) [25], and it has been reported that p-selectin and integrin aIIbß3 (GPIIb/IIIa) are two molecules that mediate the binding of platelets to human tumour cells [26–30]. Qi et al. found that p-selectin ligands were involved in tumorigenesis and development by mediating the binding of CTCs to circulating platelets [31]. In addition, studies have found that TNF-α decreased mRNA for P-selectin in human endothelial cells [32–34]. However, the specific mechanism of P-selectin in NPC has not been well clarified. This needs to be further explored and validated.

In addition, GO enrichment analysis showed that the early-stage DEGs were mainly inherent to cell junctions, while the advanced stage was mainly enriched in platelet aggregation and activation genes. KEGG analysis showed that the NF-κB signalling pathway was enriched mainly in early NPC, while platelet activation and the MAPK signalling pathway were identified as the main enriched pathways in the advanced stage. GO and KEGG analyses of co-expressed DEmRNAs showed that these DEmRNAs are inseparable from platelet activation, aggregation, degranulation, and the MAPK signalling pathway. There are differences in the biological functions of regulated genes at different stages of the development of nasopharyngeal carcinoma. Our findings may provide new insights for TEP mRNA as a marker for early diagnosis of NPC.

We analysed the gene types of the DEGs in platelets of patients with early and advanced NPC and healthy blood donors. The results showed that most of the genes in these samples belonged to protein-coding genes and lncRNAs. Therefore, the identification of DElncRNAs and DEmRNAs is a key step in understanding the involvement of lncRNA–miRNA–mRNA in the occurrence and development of nasopharyngeal carcinoma and in developing new diagnostic biomarkers. In this study, RNA-seq identified 12 up-regulated and 2 down-regulated lncRNAs in normal *versus* early-stage NPC; 33 up-regulated and 21 down-regulated lncRNAs in early-stage *versus* advanced-stage; 175 up-regulated and 102 down-regulated lncRNAs in normal *versus* advanced-stage. Many of these genes are related to cancer. For example, the long-chain non-coding RNA lncC01137 contributes to the development of oral squamous cell carcinoma and is negatively regulated by miR-22-3p [35] and up-regulated in LUAD [36]. In another example, Liu et al. found that lncRNA AP000487.1 is up-regulated in high-risk patients with oesophageal cancer and can be used as a new prognostic marker for this disease [37]. It has been reported that another lncRNA, AC147651.3, is significantly increased in the advanced stage of renal clear cell carcinoma compared with the early stage, and can be used as a potential prognostic biomarker of immune regulation [38]. The detailed information on DElncRNAs is shown in Fig. 4.

The DEmRNAs were analysed by GO and KEGG. KEGG analysis showed that target genes were significantly enriched in viral carcinogenesis. Signalling pathways related to Epstein-Barr virus (EBV) carcinogenesis include basal transcription factors, p53, MAPK, NF-κB, JAK-STAT, and B cell receptor signalling pathways. NFκB acts as a molecular hub connecting inflammation and cancer, and has been identified as a key factor in the development of malignant tumours [39]. As the typical NF-κB signal, p50/RELA has a limited function in the NPC. Therefore, the NF-κB signalling occurs through atypical pathways [40]. In addition, NF-κB signalling may also be affected by the activation of EBV-miR-BART13, which is a type of EBV miRNA. In vivo and in vitro studies have shown that these miRNAs can promote the growth and metastasis of NPC cells [41, 42]. MAPK plays a vital role in the process of cell proliferation, differentiation, transformation, and apoptosis. In osteosarcoma, FGF5 promotes cancer cell proliferation by activating the MAPK signalling pathway [43]. Studies have confirmed that in non-small cell lung cancer, exosomal miR-338-3p down-regulates CHL1 expression by inhibiting the activation of the MAPK signalling pathway, thereby inhibiting tumour cell metastasis [44]. Zhu et al. demonstrated that trans-activation promotes a malignant phenotype of NPC by targeting the BTG anti-proliferative factor 3 and activating autophagy to regulate MAPK signalling [45]. Our research shows that the MAPK and NF-κB signalling pathways play a key role in the occurrence and development of nasopharyngeal carcinoma, but the specific underlying mechanisms need to be further studied.

In recent years, lncRNA regulation has gained attention in various cancer research fields [46]. Thirty-three kinds of cancers have been confirmed to involve lncRNAs [47]. LncRNA–mRNA interaction either controls mRNA translation and degradation, or it can be a miRNA sponge [48]. Studies have shown that the lncRNA–miRNA–mRNA network also plays an important role in the occurrence and development of tumours [49]. In a 2020 study, the lncMIR4435-2HG/miR-513a-5p/KLF6 axis was studied, which up-regulated clear cell renal cell carcinoma KLF6, proven to be a typical inhibitor of various localized tumours, including prostate and colorectal [50–54]. Zhang et al. confirmed that lncRNA BANCR expression is significantly up-regulated in gastric cancer, which can promote the expression of NF-κB1 and enhance its 3' UTR activity. Meanwhile, miRNA-9 (miR-9) targets NF-κB1 to enhance its function. NF-κB1 and miR-9 are involved in the process of BANCR promoting cancer cell proliferation and inhibiting apoptosis [55]. Yuan et al. found that lncRNA ATB activated by TGF-β in hepatocellular carcinoma can competitively bind the mir-200 family, up-regulate ZEB1 and ZEB2, and induce EMT and early invasion. In addition, the interaction between lncRNA ATB and IL-11 mRNA triggers the STAT3 signal, which can promote tumour cell colonization in organs [56]. In cholangiocarcinoma, lncRNA SNHG3 can competitively adsorb miR-3173-5p (a tumour suppressor miRNA), release its inhibitory effect on the oncogene ERG, and promote the malignant phenotype of cancer cells [57].

Our findings are consistent with those of the previous research. Our lncRNA–miRNA–mRNA ceRNA network shows that SELP is regulated by five lncRNAs through four corresponding miRNAs. The miRNA binds to the 3′ UTR region of lncRNA and mRNA through base complementary pairing. Through bioinformatic analysis, we found that these DElncRNAs and SELP are down-regulated in nasopharyngeal carcinoma. SELP expression presents small differences in the early stages that increase in the advanced, indicating that the difference in the expression of this gene gradually grows with the progression of cancer. Therefore, it seems that the down-regulation of SELP can promote the occurrence and development of NPC. This gene not only can be used as a potential early diagnosis biomarker for NPC, but also as an auxiliary diagnostic index and potential therapeutic target for the staging of this disease.

Our research, however, has certain limitations. First, we did not conduct further experimental verification. In the future, we will collect more samples to identify the specific protein of SELP in NPC and conduct a series of wet labs to verify the bioinformatics analyses, such as double luciferase gene reporter assay to verify the binding relationship between lncRNA–miRNA–mRNA. Quantitative real-time PCR or western blot analysis were used to verify the predicted genes and signalling pathways, and to reveal the underlying mechanisms in the early stage of NPC. Second, the sample size is relatively small and needs to be expanded, as large-scale studies provide stronger evidence. Finally, the machine learning algorithm needs to be optimized, as its accuracy and clinical stability are still lacking.

In summary, we have identified five DElncRNAs and one DEmRNA through bioinformatic analysis. The MAPK and NF-κB signalling pathways may play an important role in the pathogenesis of nasopharyngeal carcinoma. We have also constructed a lncRNA–miRNA–mRNA network to better understand the underlying biological mechanisms between the identified genes. These findings may provide new insights into NPC pathogenesis and help explore biological markers for the early diagnosis of this kind of cancer.

## Conclusion

Through bioinformatic technology, we identified DElncRNAs and DEmRNA, constructed the lncRNA–miRNA–mRNA network, and predicted the biological processes and signalling pathways of DEGs in the early and advanced stages of nasopharyngeal carcinoma. In this study, we believe that SELP may be used as a potential blood biomarker for early diagnosis of NPC. Our research may provide new insights for exploring the biological mechanisms of NPC and potential biomarkers for its early diagnosis. However, the specific early molecular mechanisms of NPC still need further experimental verification.

## Supplementary Information

Below is the link to the electronic supplementary material.

Supplementary file1 (DOCX 4085 KB)

## Data Availability

Not applicable.

## Abbreviations

cDNA: Complementary DNA
ceRNA: Competing endogenous RNA
cfDNA: Circulating free DNA
CTC: Circulating tumour cells
CtDNA: Circulating tumour DNA
DEG: Differentially expressed gene
DElncRNA: Differentially expressed long non-coding RNA
DEmRNA: Differentially expressed messenger RNA
GEO: Gene expression database
GO: Gene ontology
KEGG: Kyoto Encyclopedia of Genes and Genomes
lncRNA: Long non-coding RNA
miRNA: Micro RNA
mRNA: Messenger RNA
NPC: Nasopharyngeal carcinoma
PCA: Principal component analysis
RNAi: RNA interference
