# Supplementary material for: Prediction of Potential Biomarkers in Early-Stage Nasopharyngeal Carcinoma Based on Platelet RNA Sequencing
Source: Mol Biotechnol. 2022 Nov 29;65(7):1096–108. doi: 10.1007/s12033-022-00611-z (PMC10271896; doi:10.1007/s12033-022-00611-z)
Supplement: Supplementary file 1 — Supplementary file1 (DOCX 4085 KB) [file 12033_2022_611_MOESM1_ESM.docx]

Additional files:

Additional file 1: Fig. S1. The DElncRNA-DEmRNA interaction network in nasopharyngeal carcinoma (NPC). The correlation coefficient of the DElncRNA-DEmRNA pair shown in the network diagram is > 0.7, and the correlation p value is < 0.05.

Additional file 2: Fig. S2. Scatter plot of lncRNA-mRNA relationships of six platelet-regulated genes.

Additional file 3: Fig. S3. ceRNA network diagram of lncRNA -miRNA-mRNA. Predictive analysis diagram of the targeting relationship between DElncRNAs and DEmRNAs in Fig. 5A and miRNA.

Additional file 1: Fig. S1. The DElncRNA-DEmRNA interaction network in nasopharyngeal carcinoma (NPC). The correlation coefficient of the DElncRNA-DEmRNA pair shown in the network diagram is > 0.7, and the correlation p value is < 0.05.

Additional file 2: Fig. S2. Scatter plot of lncRNA-mRNA relationships of six platelet-regulated genes.

Additional file 3: Fig. S3. ceRNA network diagram of lncRNA -miRNA-mRNA. Predictive analysis diagram of the targeting relationship between DElncRNAs and DEmRNAs in Fig. 5A and miRNA.
